# Resistance of *Acta2*^*R149C/+*^ mice to aortic disease is associated with defective release of mutant smooth muscle α-actin from the chaperonin-containing TCP1 folding complex

**DOI:** 10.1016/j.jbc.2021.101228

**Published:** 2021-10-01

**Authors:** Jiyuan Chen, Kaveeta Kaw, Hailong Lu, Patricia M. Fagnant, Abhijnan Chattopadhyay, Xue Yan Duan, Zhen Zhou, Shuangtao Ma, Zhenan Liu, Jian Huang, Kristine Kamm, James T. Stull, Callie S. Kwartler, Kathleen M. Trybus, Dianna M. Milewicz

**Affiliations:** 1 Division of Medical Genetic, Department of Internal Medicine, McGovern Medical School, University of Texas Health Science Center at Houston, Texas, USA; 2 Department of Molecular Physiology and Biophysics, University of Vermont, Burlington, Vermont, USA; 3 Department of Physiology, University of Texas Southwestern Medical Center, Dallas, Texas, USA

**Keywords:** aortic disease, smooth muscle actin, actin folding, *ACTA1*, gene for skeletal muscle α-actin, *ACTA2*, gene for smooth muscle α-actin, CCT, chaperonin-containing TCP-1, HTAD, heritable thoracic aortic disease, L-NAME, l-Nω-nitroarginine methyl ester, MRTF-A, myocardin-related transcription factor-A, PE, phenylephrine, RLC, regulatory light chain, SM, smooth muscle, SMC, smooth muscle cell, TAC, transverse aortic constriction, TIRF, total internal reflection fluorescence

## Abstract

Pathogenic variants of the gene for smooth muscle α-actin (*ACTA2*), which encodes smooth muscle (SM) α-actin, predispose to heritable thoracic aortic disease. The *ACTA2* variant p.Arg149Cys (R149C) is the most common alteration; however, only 60% of carriers have a dissection or undergo repair of an aneurysm by 70 years of age. A mouse model of *ACTA2* p.Arg149Cys was generated using CRISPR/Cas9 technology to determine the etiology of reduced penetrance. *Acta2*^*R149C/+*^ mice had significantly decreased aortic contraction compared with WT mice but did not form aortic aneurysms or dissections when followed to 24 months, even when hypertension was induced. *In vitro* motility assays found decreased interaction of mutant SM α-actin filaments with SM myosin. Polymerization studies using total internal reflection fluorescence microscopy showed enhanced nucleation of mutant SM α-actin by formin, which correlated with disorganized and reduced SM α-actin filaments in *Acta2*^*R149C/+*^ smooth muscle cells (SMCs). However, the most prominent molecular defect was the increased retention of mutant SM α-actin in the chaperonin-containing t-complex polypeptide folding complex, which was associated with reduced levels of mutant compared with WT SM α-actin in *Acta2*^*R149C/+*^ SMCs. These data indicate that *Acta2*^*R149C/+*^ mice do not develop thoracic aortic disease despite decreased contraction of aortic segments and disrupted SM α-actin filament formation and function in *Acta2*^*R149C/+*^ SMCs. Enhanced binding of mutant SM α-actin to chaperonin-containing t-complex polypeptide decreases the mutant actin *versus* WT monomer levels in *Acta2*^*R149C/+*^ SMCs, thus minimizing the effect of the mutation on SMC function and potentially preventing aortic disease in the *Acta2*^*R149C/+*^ mice.

The natural history of root or ascending thoracic aortic aneurysms is to asymptomatically enlarge over time, which weakens the aorta and predisposes to an acute aortic dissection (termed Stanford type A dissection) (1). Genetic variants, along with hypertension and a bicuspid aortic valve, are common risk factors for thoracic aortic disease (1). Up to 20% of patients with thoracic aortic disease have family history of the disease, termed heritable thoracic aortic disease (HTAD) (2, 3). Analysis of pedigrees indicates that HTAD is primarily inherited in families as an autosomal dominant condition with decreased penetrance. Currently, 11 genes are validated to cause thoracic aortic disease when altered, including the gene for smooth muscle α-actin (*ACTA2*) (4, 5). The vast majority of *ACTA2* mutations are missense mutations or protein truncations in which the transcripts do not undergo nonsense mediated decay, that is, a mutant actin monomer is produced (6). The most common recurrent *ACTA2* variant predisposing to HTAD is alteration of arginine 149 to a cysteine (R149C), responsible for disease in 24% of *ACTA2* mutation carriers (6). In contrast to other HTAD genes, *ACTA2* mutations confer particularly low penetrance, for example, only 60% of *ACTA2* R149C carriers have an aortic event (defined as repair of an aneurysm or an acute aortic dissection) by 70 years of age (6). Notably, mutation carriers that do not get aortic disease instead have early onset coronary artery disease and rarely do carriers get both vascular diseases. The etiology of the pleiotropic vascular diseases associated with this *ACTA2* mutation is unknown.

*ACTA2* encodes the smooth muscle (SM)-specific isoform of α-actin, which polymerizes to form the thin filaments of the smooth muscle cell (SMC) contractile unit. Molecular characterization of disease-causing *ACTA2* missense variants (p.R258C and p.R179H) determined that these mutations alter actin by decreasing filament stability, enhancing susceptibility to filament severing by cofilin, enhancing affinity for profilin, and slowing the speed at which SM myosin moves the mutant actin filament (7, 8, 9). *Acta2*^*−/−*^ mice were initially studied to identify a mechanism by which *ACTA2* mutations lead to thoracic aortic disease. *Acta2*^*−/−*^ mice are hypotensive and have decreased contraction of aortic segments in response to agonists but have normal vascular development and life expectancy (10). Despite the hypotension, *Acta2*^*−/−*^ mice develop aortic root and ascending aneurysms by 6 months of age, and increasing blood pressure significantly accelerated enlargement of the aortic root and ascending aorta (11). Both aortic tissue and explanted SMCs from *Acta2*^*−/−*^ aortas show increased levels of reactive oxygen species, which activates nuclear factor-κB signaling and increases angiotensin II receptor type Ia expression, thus potentiating angiotensin II signaling in vascular SMCs without an increase in local angiotensin II levels. Losartan, an angiotensin II receptor blocking agent, attenuated aneurysm formation, supporting that signaling through the angiotensin II receptor type Ia was in part responsible for thoracic aneurysm formation in the *Acta2*^*−/−*^ mice (11).

To confirm pathogenic pathways responsible for thoracic aortic disease in individuals with *ACTA2* mutations, the heterozygous R149C missense mutation was introduced into mice. Despite decreased force generation in the aorta and *in vitro* evidence that the mutant SM α-actin alters interaction with binding proteins, the *Acta2*^*R149C/+*^ mice do not form thoracic aortic aneurysms or dissect, even when biomechanical forces on the ascending aorta are increased. Surprisingly, our studies show that the mutant SM α-actin remains associated with chaperonin-containing TCP-1 (CCT) complex responsible for folding monomeric actins, which leads to reduced amounts of the mutant SM α-actin available for cellular functions compared with WT SM α-actin in SMCs. These results suggest that lower levels of available mutant SM α-actin compared with WT SM α-actin may underlie the decreased penetrance of thoracic aortic disease in *ACTA2* R149C patients.

## Results

### Decreased contraction of the aorta of *Acta2*^*R149C/+*^ mice

**Figure 1:**
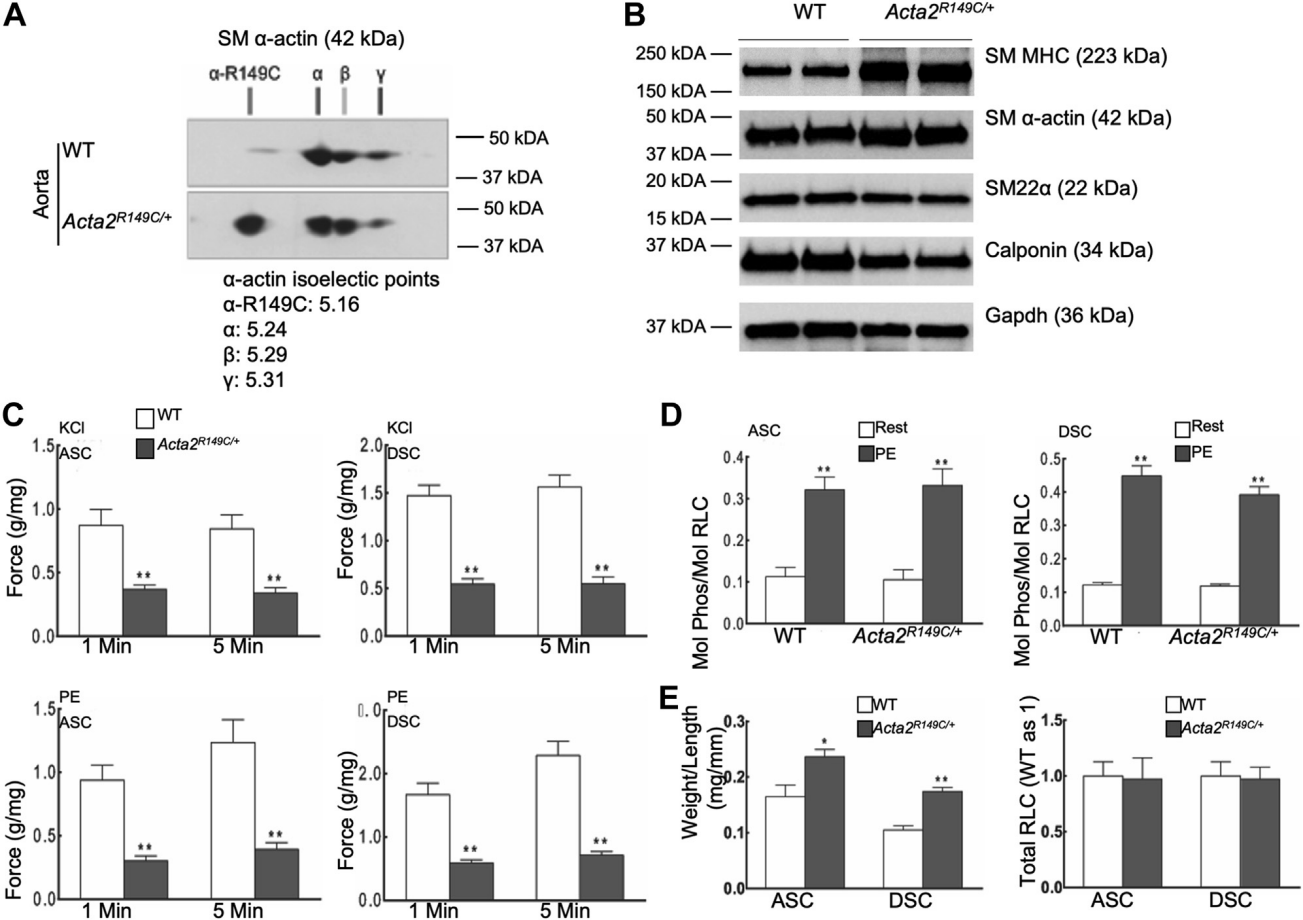
**Force development is decreased in aortic tissues from *Acta2***^***+/R149C***^**mice.***A*, two-dimensional gel of lysates of WT and *Acta2*^*R149C/+*^ aorta with pan-actin antibody confirms the production of R149C SM α-actin in the aorta of *Acta2*^*R149C/+*^ mice at 2 months of age (n = 3 mice per genotype). *B*, Western blot analysis of aortic tissue lysates shows increased amounts of smooth muscle myosin heavy chain (SM MHC) but decreased amounts of calponin, whereas there was no change in SM22α and SM α-actin in the aortas of *Acta2*^*R149C/+*^ mice compared with WT mice at 2 months of age (n = 6 per genotype). *C*, KCl (65 mM) or phenylephrine (PE, 10 μM)-induced force generation of aortic rings from both ascending (ASC) and descending (DSC) thoracic aorta of *Acta2*^*R149C/+*^ mice was significantly decreased during the initial (1 min) or sustained (5 min) contraction phases compared with the WT mice at 2 months of age. Force measurements were normalized as grams of developed force per tissue wet weight. ∗∗*p* < 0.01, compared with WT mice. n = 5 for ASC and 10 for DSC. *D*, analysis of aortic tissue lysates showed that *Acta2*^*R149C/+*^ and WT mice at 2 months of age had comparable extents of RLC phosphorylation at rest (Rest) in both ASC and DSC aorta and increased RLC phosphorylation up to 30% and 40% in ASC and DSC aorta, respectively, in response to PE (10 μM). ∗∗*p* < 0.01, compared with Rest. n = 5 for ASC and 10 for DSC. *E*, the ratio of tissue weight to length in both ASC and DSC thoracic aorta increased, but RLC total protein expression did not change in *Acta2*^*R149C/+*^ mice compared with WT mice at 2 months of age. ∗*p* < 0.05; ∗∗*p* < 0.01, compared with WT mice. n = 5 for ASC and 10 for DSC. Statistical differences between WT and mutant mice were analyzed by a Student's *t* test. RLC, regulatory light chain; SM, smooth muscle.

A mouse model of *ACTA2* p.Arg149Cys was generated by introducing the mutation into the endogenous *Acta2* allele using CRISPR/Cas9 technology in C57BL/6NJ mice and was designated as *Acta2*^*R149C/+*^ (Fig. S1*A*). No homozygous mice were born with heterozygous mating, suggesting the *Acta2*^*R149C/R149C*^ mice are not viable. Sequencing of the *Acta2* complementary DNA isolated from aortic tissue confirmed expression of the mutant and WT alleles (data not shown). To confirm production of the mutant protein, actin isoform content was analyzed by two-dimensional gel electrophoresis, followed by immunoblot analyses using a pan-actin antibody (Fig. 1*A*). Trace amounts of nonspecific bands most likely represent post-translational modification of the actin. Expression of both WT and mutant SM α-actin was confirmed in the SM-dependent organs of *Acta2*^*R149C/+*^ mice, including the aorta and bladder (Figs. 1*A* and S1*B*). Levels of contractile and SMC differentiation markers were assessed in aortic tissue lysates and showed increased levels of SM myosin heavy chain but decreased levels of calponin in mutant aorta compared with WT aorta, whereas there were no changes in SM α-actin and SM22α (Fig. 1*B* and Table S1).

The blood pressures of WT and *Acta2*^*R149C/+*^ mice at 2 months of age did not differ (mutant *versus* WT systolic blood pressure: 121 ± 3.6 mm Hg *versus* 121 ± 3.5 mm Hg, *p* = 0.93, and diastolic blood pressure: 90 ± 2.9 mm Hg *versus* 93 ± 3.9 mm Hg, *p* = 0.48; n = 5 per genotype). Force generation in aortic rings from both ascending and descending thoracic aorta from the *Acta2*^*R149C/+*^ mice was decreased compared with the WT mice (Fig. 1*C*). The KCl-induced force development, resulting from membrane depolarization, was decreased by 55 to 60% in the mutant ascending aorta and 60 to 65% in the mutant descending thoracic aorta. Similarly, the phenylephrine (PE)-induced force development acting *via* G-protein–coupled α-adrenergic receptors was decreased by 65 to 70% in both ascending and descending mutant thoracic aorta. Thus, the aortic rings from both ascending and descending thoracic aorta showed decreased force generation in *Acta2*^*R149C/+*^ mice compared with WT mice. Phosphorylation of the SM myosin regulatory light chain (RLC) initiates SMC contraction. Mice were treated with PE and assessed for differences in the extent of RLC phosphorylation in the aortic segments (Fig. 1*D*). PE treatment increased RLC phosphorylation in ascending aorta to 30% and in descending thoracic aorta to 40% in both the *Acta2*^*R149C/+*^ and WT mice. There was an increase in the ratio of tissue weight to length in both ascending and descending thoracic aorta in the *Acta2*^*R149C/+*^ mice without differences in total RLC protein expression (Fig. 1*E*). In contrast to decreased contraction observed in aortic tissues of mutant mice, where SM α-actin is the predominant actin isoform, there were no significant differences in contractile responses to KCl or carbachol or in levels of RLC phosphorylation in urinary bladder strips between mutant and WT mice (Fig. S1*C*). Together, our data indicate altered force generation in the aortas of *Acta2*^*R149C/+*^ mice with no differences in RLC phosphorylation.

### *Acta2*^*R149C/+*^ mice have increased aortic medial area but no thoracic aortic disease

**Figure 2:**
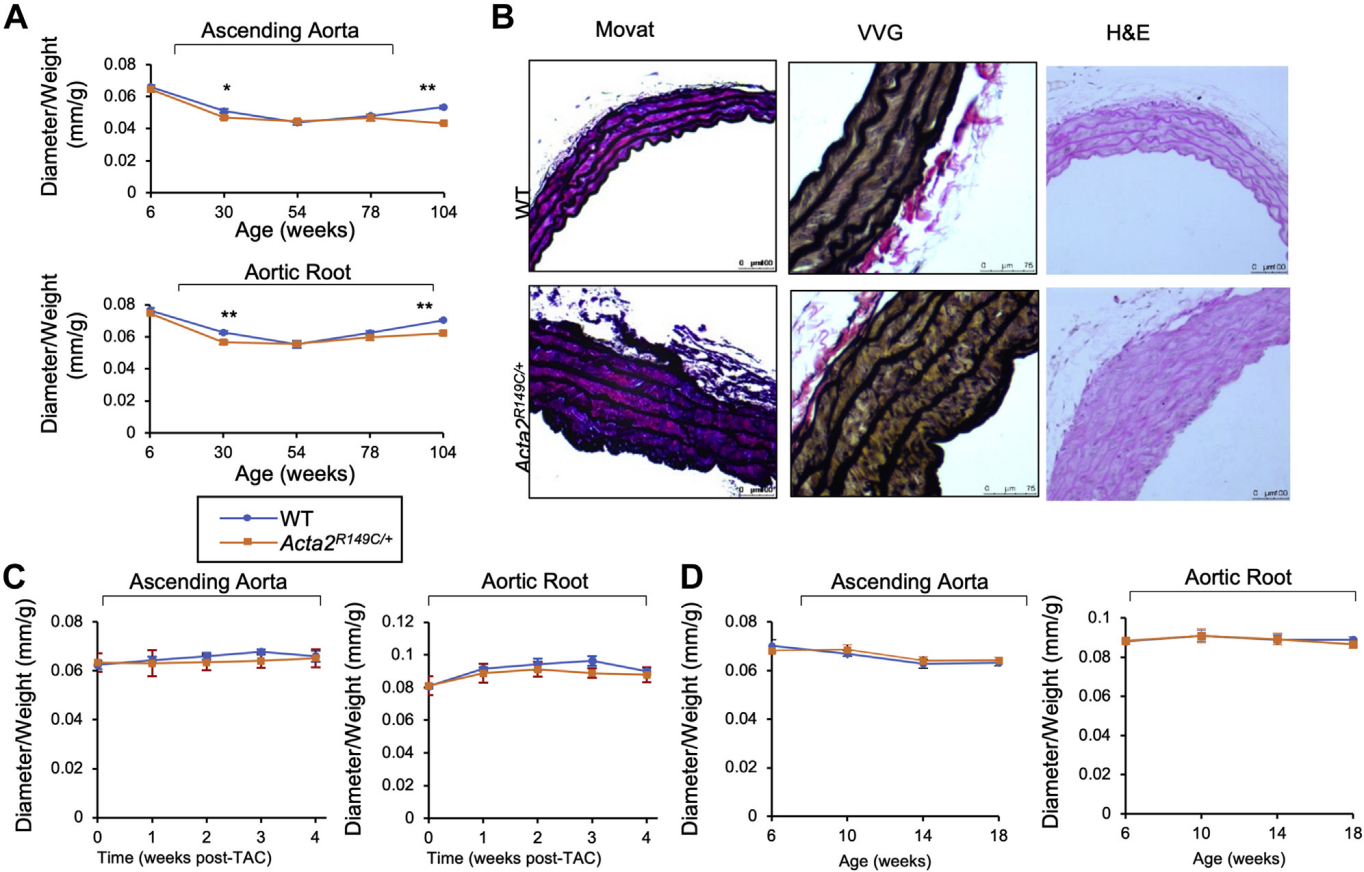
***Acta2***^***R149C/+***^**mice have increased aortic wall thickness but do not develop thoracic aortic aneurysms, even when blood pressure is increased.***A*, echocardiography analysis shows smaller size of ascending aorta and aortic root in the *Acta2*^*R149C/+*^ mice compared with WT mice in young mice, but after 30 weeks of age, the mutant and WT aortas are of the same diameter and remain similar through 104 weeks of age (*p* < 0.05, *p* < 0.01 compared with WT mice; n = 7 or 8 per group). *B*, histologic stains of aortic sections showed significantly increased aortic wall thickness with significant decrease in cell density, but there was no significant increase in elastin layers and elastin breaks in the *Acta2*^*R149C/+*^ mice at 5 months of age (*p* < 0.05, *p* < 0.01 compared with WT mice; n = 5 per group). Movat and H&E scale is 100 μm. VVG scale is 75 μm. *C*, weekly echocardiograms indicated that *Acta2*^*R149C/+*^ mice subjected to transaortic constriction (TAC) at 3 months of age had similar growth rate of both ascending aorta and aortic root to WT mice (n = 3 or 6 per group). *D*, echocardiography analysis demonstrated no aortic enlargement in *Acta2*^*R149C/+*^ mice compared with WT mice when blood pressure was elevated by administration of L-N_ω_-nitroarginine methyl ester (L-NAME; 0.3 g/l in drinking water) and high salt diet (HSD) starting at 6 weeks of age and lasting for 3 months (*p* < 0.05 compared with mice fed normal diet; n = 8 per group). Statistical differences between WT and mutant mice or mice administered with L-NAME and HSD and control mice were analyzed by a Student's *t* test or one-way ANOVA in *A*–*D*.

*Acta2*^*R149C/+*^ and WT mice have similar body weight and survival over 24 months (*p* = 0.08; Gehan–Breslow–Wilcoxon test). Echocardiography assessment of the aorta over 24 months (104 weeks) indicates that the aortic diameter of *Acta2*^*R149C/+*^ mice is initially smaller than the WT mice in young mice, but after 30 weeks of age, the mutant and WT aortas are of the same diameter and remain similar until 104 weeks of age when the mutant aorta is again smaller (Fig. 2*A*). Appraisal of the aortic pathology at 5 months of age indicated that the *Acta2*^*R149C/+*^ aortic walls are significantly thicker (*p* = 0.045), with widening of the space between the elastin lamellae, and there is a corresponding decrease in cell density (*p* = 0.0012) (Fig. 2*B* and Table 1). There is no increase in elastin breaks (*p* = 0.503) or proteoglycan deposition (*p* = 0.108).

**Table 1:** Aortic wall pathology in *Acta2*^*R149C/+*^ mice *versus* WT mice^a^

Mouse genotype	Wall thickness (mm)	Cells (×10^3^)/mm^2^	Lamellae layers	Elastin breaks
WT	0.13 ± 0.02	823.81 ± 64.02	7.8 ± 0.58	0.7 ± 0.37
*Acta2*^*R149C/+*^	0.179 ± 0.02^b^	491.75 ± 22.32^b^	8.4 ± 0.32	1.1 ± 0.43

a Data were obtained from five mice per group. Error bars indicate SD.

b *p* < 0.05.

Increased blood pressure is a major risk factor for thoracic aortic aneurysm growth and progression to acute aortic dissections in humans and mouse models, including the *Acta2*^*−/−*^ mouse model (11, 12). Therefore, we increased biomechanical forces on the ascending aorta using both surgical transverse aortic constriction (TAC) in the arch and treatment with L-N_ω_-nitroarginine methyl ester (L-NAME; Cayman Chemical) and a high salt diet. The increase in blood pressure using either method resulted in mild impairment of left ventricular systolic function in mutant mice when compared with WT mice (Figs. S2 and S3) (13). Despite the increased pressures on the ascending aorta with TAC, the aortas in the *Acta2*^*R149C/+*^ mice did not enlarge to a greater extent than the WT aortas, and no mice experienced aortic rupture (Fig. 2*C*). Similarly, treatment with L-NAME and a high salt diet increased blood pressures to the same extent in both the WT and mutant mice (Table S2). There was no difference in aortic diameter and no aortic ruptures in either WT or mutant mice treated with L-NAME and a high salt diet (Fig. 2*D*).

### Biochemical characterization of R149C SM α-actin predicts decreased force generation

We biochemically characterized the SM α-actin with the R149C mutation to better understand its physiological impact. When HIS-tagged SM α-actin R149C was expressed in the baculovirus/Sf9 cell expression system, good yields of soluble expressed protein were obtained, but no mutant actin protein bound to the HIS affinity purification column. We hypothesized that the HIS tag may be inaccessible because of retention of mutant actin inside its folding complex, the CCT complex. Supporting this conclusion is the fact that *in vitro*–translated mammalian skeletal muscle α-actin binds to but is not released from the heterologous yeast CCT complex. Altering a single amino acid residue in skeletal muscle α-actin, changing asparagine 299 to a threonine, (N299T) results in the release of skeletal muscle α-actin from the yeast CCT complex and successful *in vitro* purification (14). Based on these data, N299T was introduced into the R149C and WT SM α-actin constructs. This strategy successfully allowed the R149C/N299T SM α-actin expressed in Sf9 cells to bind to the HIS column and be purified for subsequent biophysical studies, supporting the hypothesis that retention in the Sf9 CCT prevented purification of the R149C single mutant SM α-actin.

**Figure 3:**
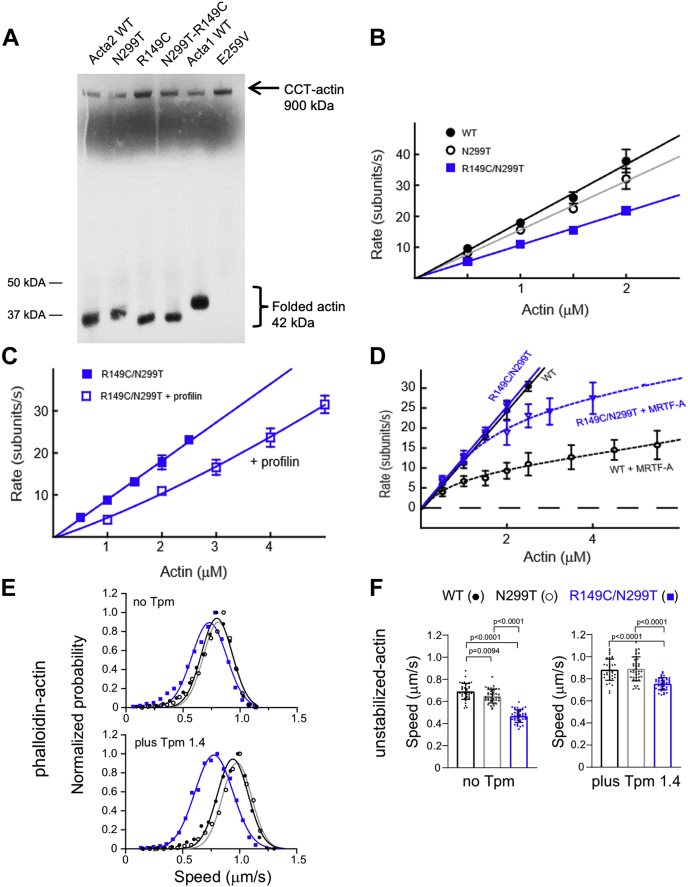
**Polymerization and motility of WT and mutant actins and their interaction with CCT and actin-binding proteins.***A*, quantitation of *in vitro* translation reaction products shows significantly increased ratio of CCT-bound:released actin for R149C actin compared with WT actin (1.14 ± 0.47 *versus* 0.42 ± 0.1, *p* = 0.01) and R149C/N299T actin (0.64 ± 0.22, *p* = 0.048). Representative gel shown from six different assays. *B*, the actin concentration dependence of the polymerization rate of WT, N299T, and R149C/N299T monomers. See Table 2 for assembly and disassembly values. *C*, the polymerization rate of R149C/N299T in the absence or the presence of 3 μM profilin yielded a dissociation constant of the mutant actin for profilin of 2.7 μM, comparable to the value of 3.0 μM previously obtained for WT (7). The curve is the fit to the data as described previously (7). *D*, the actin polymerization rate of WT and R149C/N299T in the presence of 3 μM MRTF-A. The curves are fits to the data as described previously (8). Fit to the R149C/N299T data yields a *K*_*d*_ of 3.3 μM and a Hill coefficient of 4.7, *versus* WT values of 1.8 μM and a Hill coefficient of 4.7. All WT data (*B*–*D*) are from previously published work (7, 8). *E*, Gaussian distribution of speeds at which rhodamine phalloidin–stabilized actin (WT, N299T, or R149C/N299T) is moved by phosphorylated smooth muscle myosin in the absence (*upper panel*) or the presence (*lower panel*) of tropomyosin (Tpm 1.4). Speeds of large numbers of filaments were tracked with a semiautomated program and are provided in Table S3. All pairs show statistically significant differences primarily because of the large dataset. Data were obtained using two protein preparations and two to four individual experiments. *F*, speed of movement using filaments that were not stabilized with phalloidin. Actin speeds were tracked manually and are provided in Table S3. All pairs were statistically different except for WT *versus* N299T in the presence of Tpm1.4 (*p* = 0.925). Data were obtained using two protein preparations and two individual experiments. Statistical significance was determined by one-way ANOVA followed by a Tukey's honest significant difference post hoc test. CCT, chaperonin-containing TCP-1; MRTF-A, myocardin-related transcription factor-A.

To determine if SM α-actin R149C is retained in the mammalian CCT complex, *ACTA2* WT and mutant constructs were translated using a rabbit reticulolysate system, which includes the CCT complex. The translated proteins were analyzed on nondenaturing gels, and a higher and lower molecular weight band was present, corresponding to CCT-bound and CCT-unbound SM α-actin (Fig. 3*A*). A skeletal muscle α-actin (*ACTA1*) variant, E259V, that was previously shown to have increased association with the CCT complex was used as a positive control (15). Significantly more *ACTA2* R149C monomers associated with the CCT complex than the WT or N299T actins. Introduction of the N299T mutation into the R149C construct led to the mutant actin being released from the CCT complex similar to WT. Quantitation of reaction products from six *in vitro* translation assays indicates significantly increased ratio of CCT bound to released actin for R149C actin compared with either WT actin (1.14 ± 0.47 *versus* 0.42 ± 0.1, *p* = 0.01) or R149C/N299T actin (0.64 ± 0.22, *p* = 0.048).

Using SM α-actin isolated from the baculovirus/Sf9 cell expression system, we assessed the ability of the altered SM α-actins to form filaments and interact with a subset of actin-binding proteins *in vitro* (7, 8). Because R149C is not released from the CCT complex and therefore cannot be purified, R149C/N299T SM α-actin was used for these studies. Polymerization of individual filaments in real time was followed by total internal reflection fluorescence (TIRF) microscopy, and the rate of growth of actin filaments quantified as a function of actin concentration (Fig. 3*B*). The slope of this plot is the assembly rate, the *y*-intercept is the disassembly rate, and the *x*-intercept is the critical concentration. The R149C/N299T and N299T filaments had a similar critical concentration as WT that indicates similar filament stability, although the assembly rate of R149C/N299T was reduced to ∼30% (Table 2). Filaments formed from WT, N299T, and R149C/N299T were incubated with various concentrations of cofilin to assess their susceptibility to severing. Surprisingly, N299T filaments showed extreme resistance to cofilin cleavage even as high as 600 nM cofilin (Table 3). R149C/N299T filaments showed a higher frequency of cleavage than N299T filaments, but the absolute frequency was much lower than for WT filaments. Thus, we were not able to conclusively determine how the presence of the R149C alteration affects cofilin severing of filaments.

**Table 2:** Polymerization rates of WT and mutant actins

Actin	Assembly rate (subunits·μM^−1^·s^−1^)	Disassembly rate (subunits·s^−1^)	Critical concentration (nM)
WT^a^	15.9 ± 3.4	0.7 ± 0.6	47.8 ± 44.2
N299T^b^	13.2 ± 3.6	0.1 ± 0.02	8.7 ± 0.6
R149C/N299T^c^	10.8 ± 1.5	0.4 ± 0.6	37.0 ± 54.2

Errors indicate SD.

a Data from published work (7).

b Data were obtained from two experiments using two independent protein preparations.

c Data were obtained from four experiments using two independent protein preparations.

**Table 3:** Severing frequency of WT and mutant actin filaments by cofilin

Frequency (min^−1^ μm actin^−1^ ×10^4^)	50 nM cofilin	100 nM cofilin	300 nM cofilin	600 nM cofilin
WT^a^	237.1 ± 90	259.2 ± 123.2	293 ± 160.6	149.7 ± 49.5
N299T^a^	3.6 ± 0.6	0.8 ± 2.0	2.4 ± 3.8	2.0 ± 3.3
R149C/N299T^b^	11.6 ± 18	28.7 ± 18.6	40.0 ± 21.8	45.4 ± 31.2

There were statistically significant differences between groups as determined by a one-way ANOVA for each cofilin concentration (50 nM cofilin, *p* = 3.68 × 10^−6^; 100 nM cofilin, *p* = 6.5 × 10^−6^; 300 nM cofilin, *p* = 1.6 × 10^−8^; and 600 nM cofilin, *p* =5.9 × 10^−6^).

Error bars indicate SD.

a Data were obtained from two experiments.

b Data were obtained from three experiments.

**Table 4:** Increase of WT and mutant actin filaments in the presence of formin

Actin	0 nM formin	50 nM formin
WT	1	2.4 ± 0.6
N299T	1	1.5 ± 0.5
R149C/N299T	1	5.4 ± 2.3

One-way ANOVA followed by a post hoc Tukey's honest significant difference test showed no significant differences neither between WT and N299T (*p* = 0.699) nor between WT and R149C/N299T (*p* = 0.083). The difference between N299T and R149C/N299T was significant (*p* = 0.03). Data were obtained from three experiments using three different protein preparations.

We assessed the interaction of R149C/N299T with profilin, an actin-binding protein that affects the free G-actin pool available for polymerization and found that R149C/N299T binds profilin with an affinity of 2.7 μM, comparable to the value of 3.0 μM previously obtained for WT (Fig. 3*C*) (7). In contrast, we identified that formin caused more than a 3-fold increase in filament number for R149C/N299T when compared with the N299T control (Table 4). We also assessed the interaction of the SM α-actins with myocardin-related transcription factor-A (MRTF-A), which serves as a coactivator for SMC-specific gene expression (16). In the cytoplasm, each MRTF-A molecule can bind up to five actin monomers in a cooperative fashion. R149C/N299T binds approximately 2-fold less to MRTF-A than WT (*K*_*d*_ of 3.3 μM *versus* 1.8 μm for WT, but the binding is very cooperative with a Hill coefficient of 4.7, compared with the value of three for WT α-actin. (Fig. 3*D*) (7, 8).

Finally, the interaction of the WT and mutant actin filaments with phosphorylated SM myosin to generate motion was assessed by an *in vitro* motility assay. With phalloidin-stabilized actin filaments, the R149C/N299T filaments showed slower speeds than either WT or N299T filaments, with the largest difference occurring in the presence of tropomyosin (Fig. 3*E* and Table S3). Larger reductions in speed were observed with R149C/N299T filaments that were not stabilized with phalloidin, both in the absence or the presence of tropomyosin (Fig. 3*F* and Table S3). The slower speeds with which SM myosin moves R149C/N299T actin filaments suggests that this important actin-binding partner interacts less favorably with mutant actin filaments, which could result in decreased force generation by the mutant SMCs as we observed *in vivo* previously.

### Altered SMC phenotype and decreased mutant SM α-actin levels in *Acta2*^*R149C/+*^ SMCs

**Figure 4:**
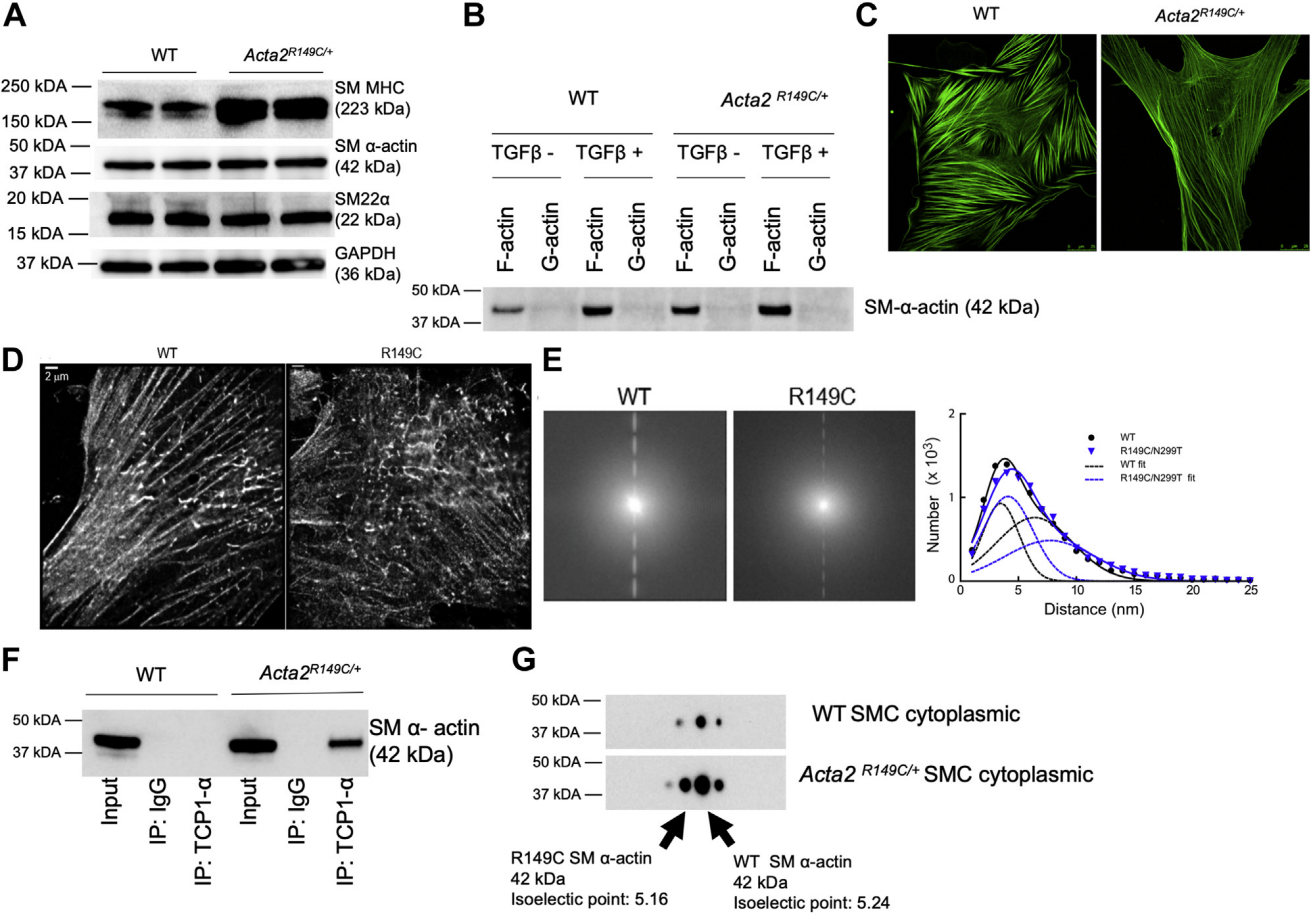
**Characterization of WT and *Acta2***^***R149C/+***^**cells.** SMCs were explanted from the ascending aorta of WT and *Acta2*^*R149C/+*^ mice. *A*, immunoblot analyses of contractile proteins showed similar amount of SM22α and SM α-actin but increased amounts of SM MHC in *Acta2*^*R149C/+*^ SMCs. Scale bars represent 25 μm. *B*, ultracentrifugation was used to separate F-actin (pellet) from G-actin (supernatant). There was no difference in F-actin (polymerized) with little or no G-actin (monomeric) in both mutant and WT SMCs. *C*, immunofluorescent staining of SMCs using a SM α-actin antibody showed that *Acta2*^*R149C/+*^ SMCs assemble SM α-actin filaments that were longer and less robust than WT SMCs. *D*, STORM images of cells expressing WT (*left*) or R149C (*right*) actin. *E*, *left*, the Fourier transform of the images showed many high-frequency bands for the WT cell. *Right*, histogram of the distance of each actin molecule's closest neighbor in WT and R149C cells. The *solid lines* are the sum of two Gaussian peaks for WT (*black*) and R149C (*blue*). The *dashed line* shows individual Gaussian peaks for WT (3.5 ± 2.2 and 6.4 ± 4.6 nm) and R149C (4.1 ± 3.0 and 7.7 ± 5.5 nm). *F*, the CCT complex was immunoprecipitated using antibodies directed against CCT1 followed by immunoblot analyses for SM α-actin, revealing increased SM α-actin associated with the CCT complex in mutant SMCs and no SM α-actin associated with the CCT complex in WT SMCs. *G*, 2D gel electrophoresis showed decreased R149C α-actin content compared with WT α-actin in the *Acta2*^*R149C/+*^ SMCs. CCT, chaperonin-containing TCP-1; MHC, myosin heavy chain; SMC, smooth muscle cell.

To further assess how the R149C SM α-actin disrupts cellular function, SMCs were explanted from the ascending aorta of the *Acta2*^*R149C/+*^ and WT mice. *Acta2*^*R149C/+*^ SMCs are differentiated to the same extent as WT SMCs based on similar SMC contractile proteins (Fig. 4*A* and Table S1). Furthermore, SM α-actin is in polymerized (F-actin) state with little to no monomeric actin (G-actin) present in either mutant or WT SMCs (Fig. 4*B*). Transforming growth factor beta treatment appropriately increases F-actin levels. Immunofluorescence also shows that *Acta2*^*R149C/+*^ SMCs assemble SM α-actin filaments, but the filaments appear longer and less robust than filaments in WT SMCs (Figs. 4*C* and S4). Super-resolution microscopy confirmed an altered actin filament structure in the *Acta2*^*R149C/+*^ SMCs (Fig. 4*D*). Actin filaments in the *Acta2*^*R149C/+*^ cell appeared more dispersed and less organized with fewer stress fiber/bundle-like structures. The mutant SMCs had filaments that aligned in all directions and covered the cell more uniformly, leading to a less oriented network than in the WT SMCs. Fourier transform of the images shows many high-frequency bands for the WT SMC that are not present in the *Acta2*^*R149C/+*^ SMCs, indicating more ordered actin structures in the WT SMCs (Fig. 4*E*, *left*). Approximately 9 million actin molecules were identified in both WT and *Acta2*^*R149C/+*^ SMCs, consistent with similar levels of actin expression. The distribution of the minimum distance between two identified actin protomers for both the WT and *Acta2*^*R149C/+*^ SMCs was fit to the sum of two Gaussian peaks. The main peak at ∼4 nm corresponds to the distance between neighboring protomers within an actin filament, and the second peak at 6 to 8 nm corresponds to either the next protomer within the same filament or an actin protomer in a neighboring filament (Fig. 4*E*, *right*). On average, the distance for the second peak fits was slightly less for WT than for the *Acta2*^*R149C/+*^ SMCs, confirming that the WT has more bundle-like structures, whereas the mutant cell actin is less organized.

Since the R149C mutant SM α-actin is retained in the CCT complex in both yeast cells and rabbit reticulolysates, we sought to determine whether there is increased retention of the R149C mutant SM α-actin in the CCT complex in *Acta2*^*R149C/+*^ SMCs. After immunoprecipitation of the CCT complex using an antibody directed against the TCP1α subunit, followed by immunoblot analyses for SM α-actin, we consistently found increased SM α-actin associated with the CCT complex in the *Acta2*^*R149C/+*^ SMCs when compared with WT SMCs (Fig. 4*F*). Based on two-dimensional gel electrophoresis, we observed that the cytoplasmic levels of mutant were lower than the WT SM α-actin in the *Acta2*^*R149C/+*^ SMCs (Fig. 4*G*).

## Discussion

Clinical data from families with the *ACTA2* R149C variant found that thoracic aortic disease was associated with significantly decreased penetrance; only 60% of carriers have aortic disease by 70 years of age (6). In comparison, *FBN1* pathogenic variants are essentially fully penetrant in individuals with Marfan syndrome (17). Here, we show that *Acta2*^*R149C/+*^ mice do not have thoracic aortic disease, thus recapitulating the decreased penetrance observed in patients with the *ACTA2* R149C mutation. The most dramatic biochemical change associated with the R149C SM α-actin was the retention of the mutant actin with the CCT complex, leading to reduced cytosolic levels of the mutant α-actin compared with WT α-actin in SMCs. Importantly, haploinsufficiency of *ACTA2* is not established to predispose to highly penetrant HTAD in humans or mice. Thus, the amount of mutant SM α-actin released from the CCT complex could potentially determine whether an individual with the *ACTA2* R149C variant has aortic disease or not.

Actins cannot fold spontaneously and require the CCT complex to achieve their native conformation (18). Previous studies have identified both skeletal and cardiac α-actin mutations that result in α-actin folding defects, such as impaired release from the CCT complex or the cochaperone, prefoldin (15, 19). For example, *ACTA1* L94P and E259V are recessive missense mutations in skeletal muscle α-actin that predispose to nemaline myopathy. L94P and E259V α-actin bind to the CCT complex and prefoldin, respectively, with such affinity that none of the mutant α-actin is released from these complexes (15). Patients homozygous for these *ACTA1* mutations have severe nemaline myopathy, but heterozygous parents are unaffected. These data indicate that individuals with heterozygous *ACTA1* alterations, leading to no mutant α-actin released from the CCT complex, have half normal levels of WT skeletal α-actin and no myopathy. Other *ACTA1* missense mutations are not sequestered in the CCT complex, and a mutant protein is produced, leading to myopathy in the heterozygous state. These data indicate that individuals with heterozygous *ACTA1* L94P and E259V lack release of the mutant α-actin from the CCT complex and prefoldin, and half normal levels of WT skeletal α-actin do not cause myopathy. Based on these data, we hypothesize that the amount of *ACTA2* R149C variant released from the CCT complex could determine the penetrance of aortic disease in both mice and humans with this variant.

We previously found that a rare *MYH11* missense variant, R247C, alters myosin function and decreases contraction of aortic segments but does not lead to thoracic aortic disease in mice (20). When this *MYH11* variant was crossed with *Acta2*^*−/−*^ mice, aortic dilation in the double mutant mouse model was augmented when compared with the *Acta2*^*−/−*^ mice alone (21). The degree in the decrease of aortic ring contraction did not correlate with rate of aortic enlargement in these studies. Thus, variants in genes triggering HTAD can disrupt protein function and aortic contraction but not lead to thoracic aortic disease. As mentioned previously, we hypothesize that there is a threshold for the levels of mutant α-actin that triggers thoracic aortic disease, but our data indicate that the threshold may not directly correlate with further decreases in aortic contraction. In the case of patients with the *ACTA2* R149C alteration, our data suggest that a factor contributing to the penetrance of thoracic aortic disease may be the degree of disruption of actin filaments by varying amounts of mutant SM α-actin released from the CCT complex. Additional genetic studies of patients with *ACTA2* R149C pathogenic variants may identify novel genetic variants that modulate the penetrance of aortic disease in these patients and provide insight into factors that alter the release of the mutant α-actin from the CCT complex.

A second mutation N299T in the R149C actin was introduced to allow the release from the CCT folding complex, and the R149C/N299T actin was thus used to assess the defects in biochemical properties caused by the R149C mutation in comparison with WT and N299T actin. In contrast to other mutant actins we have studied, R149C/N299T formed stable filaments, bound normally to profilin, and co-operatively bound to MRTF-A (7, 8). A previously studied *ACTA2* mutation, R179H, had a severe polymerization defect with a critical concentration ∼40-fold higher than WT, whereas another mutation, R258C, formed only slightly less stable filaments. Both these mutant SM α-actins were released from the CCT complex at levels similar to WT SM α-actin. A feature common to both R258C and R179H actin was their enhanced affinity for profilin, which was not shared by R149C/N299T. The poorly polymerizing R179H showed little co-operative binding to MRTF-A. Interaction of R149C/N299T with the actin-nucleator formin increased filament number to a larger extent than the N299T control. Binding of formin to R179H actin, in contrast, suppressed nucleation and slowed polymerization rates. The actin filaments in the *Acta2*^*R149C/+*^ cell appear more dispersed with less stress fiber/bundle-like structures and a less oriented appearance compared with filaments in WT cells. The hyperactive nucleation of R149C monomers by formin may enable filament formation at more dispersed locations compared with WT, which in turn would decrease bundling and possibly decrease the ability to generate force. A common feature of all three mutant actins is an impaired interaction with SM myosin, which predicts reduced contraction rate and force generation. Consistent with the *in vitro* data, *ex vivo* analyses of contractility in aortic rings confirm decreased force generation by the *Acta2*^*R149C/+*^ aortas.

The eukaryotic CCT complex folds approximately 10% of the cytosolic proteome, including cytoskeletal proteins such as tubulins and actins (18). Previous studies have identified β-tubulin mutations and β-actin mutations that result in defective folding or impaired release from the CCT complex (22). Actin is a highly conserved protein, and G146 and G150 are completely evolutionarily conserved glycine residues in the actin family that are located in the hinge, linking the small and large domains of β-actin (23). When these residues are mutated to proline in β-actin, the actin is retained in the CCT, as confirmed *via in vitro* transcription/translation assays in reticulocyte lysates (22). This study concluded that G146 and G150 are key components of the hinge around which the actin subdomains rotate during CCT folding, as confirmed by the fact that mutating these residues causes arrest of actin on the CCT complex. Previous studies have identified potential CCT interaction sites of actin including actin peptides 141 to 150 (18). It is relevant to note that *ACTA2* R149 lies within the hydrophobic cleft and may be involved in CCT–actin interactions (24).

To further assess for aortic disease in the mutant mice, we increased biomechanical forces on the ascending aorta using both TAC and treatment with L-NAME and a high salt diet. While increasing these forces accelerated aortic enlargement in *Acta2*^*−/−*^ mice, the *Acta2*^*R149C/+*^ mice did not develop aortic disease. Both methods resulted in mild impairment of left ventricular systolic function in mutant mice when compared with WT mice. We noted a transient decrease in ejection fraction in hypertensive WT mice treated with L-NAME at 14 weeks and a slower sustained decrease in ejection fraction of WT TAC mice compared with mutant TAC mice with transient increases in stroke volume and cardiac output. These varying responses to biomechanical stress may reflect a difference in fibrotic remodeling of the heart in mutant *versus* WT mice, which will be assessed in future studies.

Our data support that the presence of the R149C SM α-actin in the *Acta2*^*R149C/+*^ mice decreases aortic contraction but does not cause thoracic aortic disease, which reflects the decreased penetrance of thoracic aortic disease in patients with this *ACTA2* variant. The impaired release of mutant SM α-actin from the CCT complex decreases the level of mutant to WT SM α-actin in the cytoplasm, and clinical studies indicate that *ACTA2* variants resulting in a mutant protein are more likely to be disease-causing variants than variants causing haploinsufficiency, thus affirming that the amount of mutant actin produced is a major factor in determining whether an individual has disease or not. These data therefore provoke a hypothesis that the amount of R149 SM α-actin released from the CCT may determine whether the mutant SM α-actin causes thoracic aortic disease or not in a given individual. Since *ACTA2* R149C mutation carriers have either thoracic aortic disease or early onset coronary artery disease, the unanswered question is whether the increased retention of the mutant SM α-actin in the CCT complex conversely increases the risk for coronary artery disease.

## Experimental procedures

Additional information on methodologies is presented in the supporting information.

### Engineering and maintenance of the *Acta2*^*R149C/+*^ mice

All mouse experiments were approved and performed in accordance with institutional guidelines set forth by the Institutional Animal Care and Use Committee for the University of Texas Health Science Center at Houston (UTHealth; AWC-18-0173) and the National Institutes of Health Guidelines for the care and use of laboratory animals. CRISPR/Cas9 genome editing was used to introduce the *ACTA2-*R149C mutation into C57BL/6NJ mice (The Jackson Laboratory; catalog no. 005304).

### Preparation of SM strips from mice and force measurements

Mice were euthanized by exposure to an anesthetic, 2.5% avertin (450 mg/kg intraperitoneal), one time only for a terminal procedure (the dissection and harvesting of ascending aorta). The aorta was isolated with endothelial cells removed by gentle swabbing and excessive adventitia removed by dissection. Ascending (2–3 mm) and descending thoracic (5 mm) aortic segmental rings were mounted by triangular wires to isometric force apparatus as previously described (20). Aortic rings were passively stretched to 1.8 to 2.0 g and remained quiescent for 60 min before precontraction with 65 mM KCl in Krebs–Ringer solution. Aortic rings were also treated with 10 μM PE, an α-adrenergic agonist. Force measurements were normalized as grams of developed force per tissue wet weight.

The urothelium and adventitia were removed from isolated urinary bladder, and the SM layer dissected into longitudinal strips (0.5 × 0.5 × 8.0 mm) was mounted on an isometric force apparatus in physiological salt solution as previously described (20). Strips were equilibrated and stretched 1.2 times slack length. After 30 min of equilibration, the strips were precontracted with 65 mM KCl three times and then stimulated with 10 μM carbachol, a muscarinic agonist (Sigma). Force measurements were recorded isometrically by a Grass FT03 force transducer connected to Powerlab 8/SP data acquisition unit (AD Instruments). Stresses (Newtons per square meter) were calculated to normalize contraction responses to bladder tissue cross-sectional areas.

### Measurement of myosin RLC phosphorylation

Isolated tissues were snap frozen by clamps prechilled in liquid nitrogen for protein phosphorylation measurements. Frozen muscles were processed as described previously (20). Muscle proteins in 8 M urea sample buffer were subjected to urea/glycerol-PAGE at 400 V for 80 min to separate nonphosphorylated and monophosphorylated RLC. Following electrophoresis, protein was transferred to polyvinylidene difluoride membranes and probed with antibodies against SM myosin RLC. The ratio of monophosphorylated RLC to total RLC (nonphosphorylated plus phosphorylated) was determined by quantitative densitometry and expressed as moles of phosphate per mole of protein (20).

### Isoelectric focusing analysis

Aorta and bladder tissue samples (∼5 mg) were collected from WT and *Acta2*^*R149C/+*^ mice and homogenized directly in ReadyPrep 2-D Starter Kit Rehydration/Sample Buffer (Bio-Rad), then subjected to fractionation in a 24 cm, pH 4 to 7, immobilized pH gradient gel strip (GE Healthcare). Samples were focused for a total of 100 kVh in a PROTEAN IEF Cell (Bio-Rad). After isoelectric focusing, the portion of gel strip corresponding to pH 5.1 to 5.4 was subjected to 10% SDS-PAGE and immunoblotted using routine procedures.

### Mouse echocardiography

Echocardiography was performed with an ultrasound system (Vevo 3100 imaging system; MX550D, 40 MHz transducer; VisualSonics) at different time points indicated. Anesthesia was induced with 4% isoflurane and maintained at 1.5 to 2%; mice were then restrained supine on a heated platform to keep body temperature at 38 °C. Images were acquired and stored as a digital cine loop for offline calculations. Measurements of the aortic diameter were done in at least three separate heartbeats, and the mean of the measurements was calculated. Data analysis was performed blinded.

### Hypertensive treatment and blood pressure measurement

High salt diet (8% NaCl diet from Harlan Laboratories) and L-NAME (3.0 g/l in drinking water) were used to induce hypertension in age-matched and sex-matched *Acta2*^*R149C/+*^ and WT mice. L-NAME water was changed every day. Measurement of blood pressures in conscious mice was performed with a tail cuff blood pressure analyzer designed with volume-pressure recording technology (Model Coda; Kent Scientific Technology). Body temperature of the mice was maintained at 37 °C during measurement. Data from measurements for 3 consecutive days were used, and the mean value of the readings for each day per mouse was averaged and taken as the single blood pressure measurement for that animal.

### TAC

After initial echocardiography, mice at the age of 3 months were anesthetized with isoflurane (4% for induction and 2% for maintenance) and transferred to a heated platform. Anesthetized mice were intubated, followed by midline cervical incision, to expose the aorta. Aortic constriction was achieved by placing a 7.0 nylon suture ligature against a 27-gauge needle on transverse aorta. The needle was removed promptly to create an aortic constriction of 0.4 mm in diameter, and the chest was sealed. The mice were maintained on a heating pad for recovery. Ketoprofen (5 mg/kg subcutaneously) was administered every 24 h for 3 days after surgery. Echocardiography was performed on all surviving mice each week for 4 weeks after surgery.

### Aortic histopathology

Paraffin-embedded aorta tissue cross sections (5 μm) were stained with H&E, Verhoeff–Van Gieson, and Movat according to the standard protocols. Specimens were imaged using a Leica microscope. Quantification of aortic wall thickness was performed using ImageJ (the National Institutes of Health). Cell density and elastin breaks were quantified using the cell counter function. All quantitative analyses were performed by two or more researchers.

### SMC isolation and culture

SMCs were explanted from the ascending aortas of age-matched and gender-matched *Acta2*^*R149C/+*^ and WT littermates as previously described (https://bio-protocol.org/e2045). SMCs were maintained in SM basal medium (Promo Cell) supplemented with 20% fetal bovine serum (Gibco), insulin, epidermal growth factor, fibroblast growth factor (Promo Cell), Hepes (Millipore Sigma), sodium pyruvate (Millipore Sigma), l-glutamine (Millipore Sigma), and antibiotic/antimycotic (Millipore Sigma). SMCs were serum starved in SM basal medium containing 1% fetal bovine serum for 24 h and treated with transforming growth factor beta treatment (2 ng/ml) for 48 h unless indicated otherwise.

### Western blotting

Lysates from whole cells or tissues were fractionated by SDS-PAGE and transferred to a polyvinylidene difluoride membrane according to the standard protocols. Immunoblots were quantitated with ImageJ.

### F-to-G actin assay

The fraction of F-to-G actin was assayed using the G-actin/F-actin *In Vivo* Assay Kit (Cytoskeleton).

### Subcellular fractionation

SMCs were serum starved overnight, lysed, and fractionated using the CelLytic NuCLEAR Extraction Kit (Sigma; NXTRACT-1KT).

### Immunofluorescence

Cells were seeded on 22 mm cover slips and attached overnight. Cells were fixed in 4% paraformaldehyde in 0.1 M PBS. The SMCs were permeabilized with 0.3% Triton X-100 for 15 min, blocked in 1% bovine serum albumin in PBS for 1 h, and then incubated with α-actin primary antibody at 4 ^°^C overnight. Cells were incubated with Alexa Fluor 488 Goat-antimouse at room temperature for 1 h and Texas Red-X phalloidin for 40 min. Immunofluorescent images were obtained using the Leica DMi8 SPE confocal microscopy at the indicated magnification.

### Immunoprecipitation

Cells were lysed in 20 mM Tris, pH 8, 137 mM NaCl, 1% NP-40, and 2 mM EDTA and incubated in 10 mM Tris (pH 8), 150 mM NaCl, 0.1 mM EGTA, 20% glycerol, and 0.2% NP-40 and TCP1α antibody overnight at 4 °C with normal rabbit immunoglobulin G used as the control. Protein A/G magnetic beads were washed in 10 mM Tris, pH 8, 150 mM NaCl, 1% NP-40 three times, and incubated with samples at 4 °C for 3 h. Protein was eluted in 2× Laemmli buffer (Bio-Rad) by heating the beads at 70 °C for 10 min and collecting the eluate using magnetic separation.

### Super-resolution microscopy

Super-resolution images were obtained on a commercial Nikon N-STORM super-resolution microscope. Raw images were processed with the ImageJ plugin ThunderStorm to obtain the final super-resolution images (25). See supporting information for additional details.

### Actin cloning and expression

The base SM α-actin–thymosin construct described previously was recloned into pFastBac (Thermo Fisher Scientific) and mutated to contain either N299T or R149C/N299T (7, 8, 9). Recombinant baculovirus was produced using the Bac-to-Bac Baculovirus expression system (Thermo Fisher Scientific). See supporting information for additional details.

### Reticulocyte lysate *in vitro* translation assays

*ACTA2* and *ACTA1* were each cloned into the vector CMVTnT (Promega; catalog no. L5620) behind the T7 promoter. An additional 30 polyA tail was added after each gene for mRNA stability. All DNA preps in the reticulocyte study were eluted in RNase-free water. The SM α-actin variants introduced by site-directed mutagenesis were R149C, N299T, and R149C/N299T. All constructs were sequenced to verify mutagenesis and confirm the absence of PCR-induced errors. The individual SM α-actin variants were expressed *in vitro* in reticulocyte lysates using the Promega Transcription Translation Reaction Protocol (400 ng DNA) and labeled with ^35^S methionine. Reaction products were analyzed on 4.5% nondenaturing Safer gels with ATP and autoradiography to determine the protein levels of CCT-bound actin (26).

### Biochemical assays of actin

*In vitro* motility and TIRF microscopy assays were previously described (7, 8). TIRF microscopy used for the polymerization assays in the absence or the presence of actin-binding proteins was carried out on a Nikon ECLIPSE Ti microscope with through-objective type TIRF. The speed at which rhodamine phalloidin–stabilized actin filaments were moved by phosphorylated SM myosin was determined using a semiautomated tracking program that allowed analysis of large numbers of filaments, which were then fitted to a Gaussian distribution as previously described (7). For speed of movement using filaments that were not stabilized with phalloidin, WT filaments were formed from 25% rhodamine-labeled WT actin and 75% unlabeled WT. N299T and N299T/R149C filaments were formed from 25% rhodamine-labeled N299T actin and 75% of unlabeled N299T or N299T/R149C actin. Filament speed was tracked manually with ImageJ because the automated program could not detect the dimmer filaments. See supporting information for additional details.

### Statistical analysis

All data are representative of at least three separate experiments unless noted otherwise. Difference in survival was determined by a Gehan–Breslow–Wilcoxon test. A two-tailed unpaired *t* test or one-way ANOVA followed by a Tukey's honest significant difference post hoc test was used to determine statistical significance between groups. A *p* value less than 0.05 indicated statistical significance.

## Data availability

The data underlying this article are available from the corresponding author upon reasonable request.

## Supporting information

This article contains supporting information (7).

## Conflict of interest

The authors declare that they have no conflicts of interest with the contents of this article.
