# Supplementary material for: Resistance of Acta2R149C/+ mice to aortic disease is associated with defective release of mutant smooth muscle α-actin from the chaperonin-containing TCP1 folding complex
Source: J Biol Chem. 2021 Oct 1;297(6):101228. doi: 10.1016/j.jbc.2021.101228 (PMC8633019; doi:10.1016/j.jbc.2021.101228)
Supplement: Supplementary file 1 — Supporting information [file mmc1.pdf]

## SUPPORTING INFORMATION

***Resistance of Acta2$^{R149C/+}$ Mice to Aortic Disease is Associated with Defective Release of Mutant Smooth Muscle α-actin from the Chaperonin-containing TCP1 folding complex***

Jiyuan Chen[1], Kaveeta Kaw[1], Hailong Lu[2], Patricia M. Fagnant[2], Abhijnan Chattopadhyay[1], Xue Yan Duan[1], Zhen Zhou[1], Shuangtao Ma[1,4], Zhenan Liu[3], Jian Huang[3], Kristine Kamm[3], James T. Stull[3], Callie S. Kwartler[1], Kathleen M. Trybus[2], Dianna M. Milewicz[1]

**Corresponding Author:**
Dianna Milewicz, MD, PhD

## Additional Methods

***Engineering and maintenance of the Acta2$^{R149C/+}$ mice*** Superovulated female mice were mated with C57BL/6NJ male mice overnight. Oviducts were collected the following morning and zygotes retrieved for microinjection. Four guide RNAs were designed and validated for cutting efficiencies by the MilliporeSigma CRISPR Product Specialist Team (MilliporeSigma, Saint Louis, MO). The knock-in mice were generated by pronuclear microinjection of the Cel 1 assay-validated guide RNA with the highest cutting efficiency, (tggacgtacaactggtaggtgg; 2.5 ng/µl), an oligo (C to T snp conversion; 5.0 ng/ul) and Cas9 endonuclease (5.0 ng/µl). A silent mutation in the intronic region was introduced in order to destroy the PAM site after the first Cas9 endonuclease cut. The microinjected zygotes were implanted into the infundibulum of 3-month-old pseudo-pregnant recipient female mice (ICRxCD-1 mix) that have been mated with vasectomized male mice (ICRxCD-1 mix). To genotype the *Acta2*$^{R149C/+}$ mice, genomic DNA from the tail was amplified and sequenced; subsequently, the DNA digested with the restriction enzyme BsrGI (*Acta2*$^{R149C}$ variant introduces a new restriction site) and assessed on agarose gels. The *Acta2*$^{R149C/+}$ knock-in mouse line was established by crossing the mutant founder mouse with C57BL/6NJ wild-type mice. Five potential off target sites identified by bioinformatics analyses were sequenced and no off-target alterations were identified.

***Subcellular Fractionation*** Aliquots of fractionated proteins were separated by SDS-PAGE followed by immunoblotting with antibodies against Histone H3 (1:1000) and Gapdh (1:1000) as nuclear and cytoplasmic markers respectively to assess the purity of the fractionation. Acetone precipitation of the cytoplasmic extract was performed and the supernatant was removed. The protein pellet was re-dissolved in sample buffer containing 8 M Urea, 2% CHAPS, 50 mM DTT, 0.2% (w/v) Bio-Lyte 3/10 ampholytes. The supernatant was collected after centrifugation and an aliquot of the supernatant was taken for 2-D analysis.

***Super-resolution microscopy*** Cells were fixed, stained with α-actin antibody and Alexa 647 Rhodamine using the immunofluorescence protocol, and air-dried for storage. Before imaging, the cells were rehydrated with imaging buffer containing 50 mM Tris, pH 8.0, 10 mM NaCl, 166.6 mM β- mercaptoethanol and oxygen scavengers (0.13 mg/ml glucose oxidase, 50 µg/ml catalase, 3 mg/ml glucose). 16000~30000 frames were captured for each condition. Briefly, the individual position of fluorophores was first identified in each frame, stage drift was corrected with cross correlation, duplicated molecule positions were removed, and the final super resolution images were rendered with the normalized Gaussian option. To obtain the distribution of the closest neighboring actin monomers, the distance between the closest neighboring actin monomer was calculated for every actin monomer from coordinates obtained by ThunderSTORM with a Matlab routine. The histograms of the distance distribution were calculated and plotted with a Matlab histogram function. To obtain the Fourier transformation of the figures, super resolution images of WT and R149C cells were Fourier transformed using Matlab fft2 function, and the magnitude of the results were plotted as images.

***Actin cloning and expression*** Infected Sf9 cells (2 x $10^9$) were harvested 3 days after infection and lysed in 50 ml of 10 mM HEPES pH 8.0, 0.5 mM $CaC1_2$, 0.3 M NaCl, 0.5 mM DTT, 0.5 mM $Na_2ATP$, and protease inhibitors (0.5mM 4-(2-aminoethy)benzene-sulfonyl fluoride hydrochloride, 5 µg/ml leupeptin, 0.5 mM tosyllysine chloromethyl ketone hydrochloride). The extract was clarified (250,000 x g for 30 min) and the soluble fraction was applied to a 10 ml column of HIS-Select nickel affinity resin (Sigma-Aldrich). Non-specifically bound contaminating proteins were eluted with 10 mM HEPES pH 8.0, 0.25 mM $CaCl_2$, 0.3 M NaCl, 0.25 mM $Na_2ATP$, 0.5 mM DTT, 1µg/ml leupeptin and 10 mM imidazole pH 8.0. Actin was eluted in the same base buffer but with 200 mM imidazole. Peak fractions were pooled, concentrated using an Amicon Ultra-4 centrifugal filter (Millipore) and dialyzed against 5 mM Tris pH 8.26 (at 4$^{o}$C),

0.2 mM $CaCl_2$, 0.5 mM DTT, 0.2 mM $Na_2ATP$, 1μg/ml leupeptin. The protein was clarified (300,000 x g for 25 min) and the thymosin-HIS tag removed by cleavage with chymotrypsin (1:50 weight ratio chymotrypsin:actin, 15 min, room temperature). The actin was separated from the thymosin-HIS tag with a Mono Q 5/50 GL column (GE Healthcare) using a gradient of 0-0.3 M NaCl in 5 mM Tris pH 8.26 (at 4°C), 0.2 mM $CaCl_2$, 0.5 mM DTT, 0.2 mM $Na_2ATP$, 1μg/ml leupeptin, followed by a step to 0.5 M NaCl. Peak fractions were pooled, concentrated using an Amicon Ultra-4 centrifugal filter (Millipore), and dialyzed against 5 mM Tris pH 8.26 (at 4°C), 0.2 mM $CaCl_2$, 0.1 mM $NaN_3$, 0.5 mM DTT, 0.2 mM NaATP, 1μg/ml leupeptin and aliquots frozen for storage at -80°C.

***Biochemical assays of actin*** Compared with previous methods, the SM myosin concentration was reduced to 100 μg/ml and the blocking agent was changed to 5 mg/ml β-casein (Sigma-Aldrich C6905) for *in vitro* motility assays [7]. For polymerization assays, TIRF microscopy samples were excited with the TIRF field of a 488-nm laser line, and emission was observed with a 525/50 filter. The fluorescence image was observed with a 100X objective and recorded on an Andor EMCCD camera (Andor Technology, South Windsor, CT) at 1 frame/s.

## Supplemental Tables and Figures

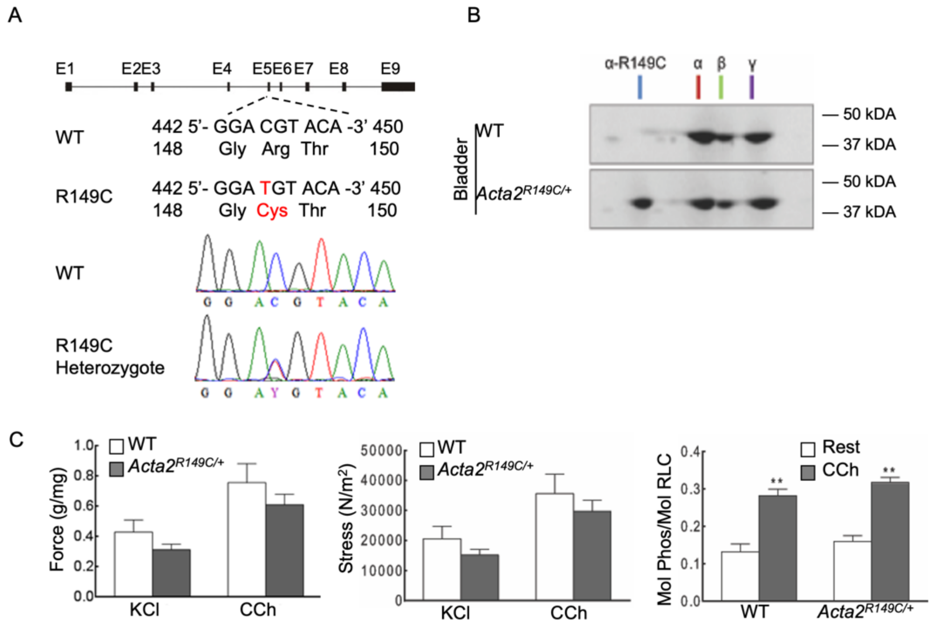


**Supplementary Figure 1: Bladder smooth muscle tissues from *Acta2*$^{R149C/+}$ mice showed no changes in contractile or RLC phosphorylation responses.** (**A**) Alignment of sequencing of genomic DNA isolated from mouse tail biopsies confirmed the heterozygous R149C mutation was present in the mice. Y indicates C/T heterozygote. (**B**) Two-dimensional (2-D) gel analysis of lysates of WT and *Acta2*$^{R149C/+}$ bladder with pan-actin antibody confirmed the production of R149C SM α-actin in the bladder of *Acta2*$^{R149C/+}$ mice at two months of age (n=3 mice per genotype). (**C**) There were no significant differences in stress responses to KCl or carbachol in isolated bladder smooth muscles between *Acta2*$^{R149C/+}$ and WT $^{mice}$. Similarly, there were no differences in RLC phosphorylation responses to carbachol at 30 sec. KCl: 65 mM KCl buffer; CCh: 10 μM carbachol. ** *P*<0.01, compared with Rest. *N*=10.

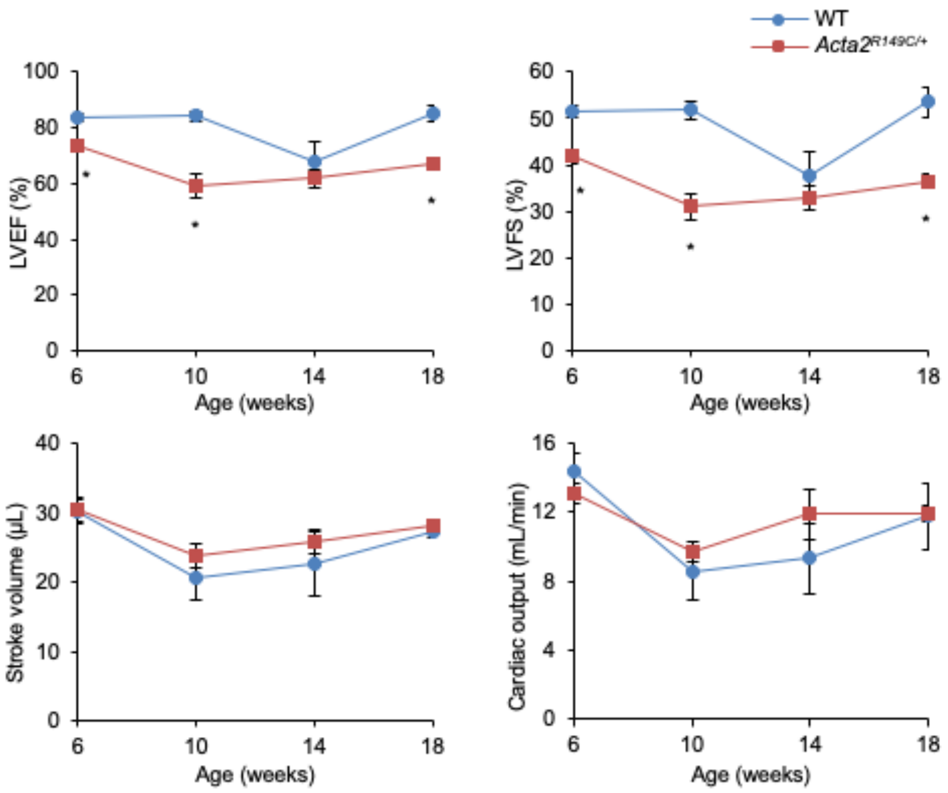


**Supplementary Figure 2: Impaired systolic function in hypertensive *Acta2*$^{R149C/+}$ mice.** Echocardiography analysis demonstrated impaired systolic function in *Acta2*$^{R149C/+}$ mice when blood pressure was elevated by administration of L-N$_{\omega}$-nitroarginine methyl ester (L-NAME, 0.3 g/L in drinking water) and high salt diet (HSD) starting at 6 weeks of age and lasting for 3 months (P< 0.05 compared to WT mice. n=8 per group). Stroke volume and cardiac output remained similar between WT and mutant mice. LVEF, Left Ventricular Ejection Fraction; LVFS, Left Ventricular Fractional Shortening.

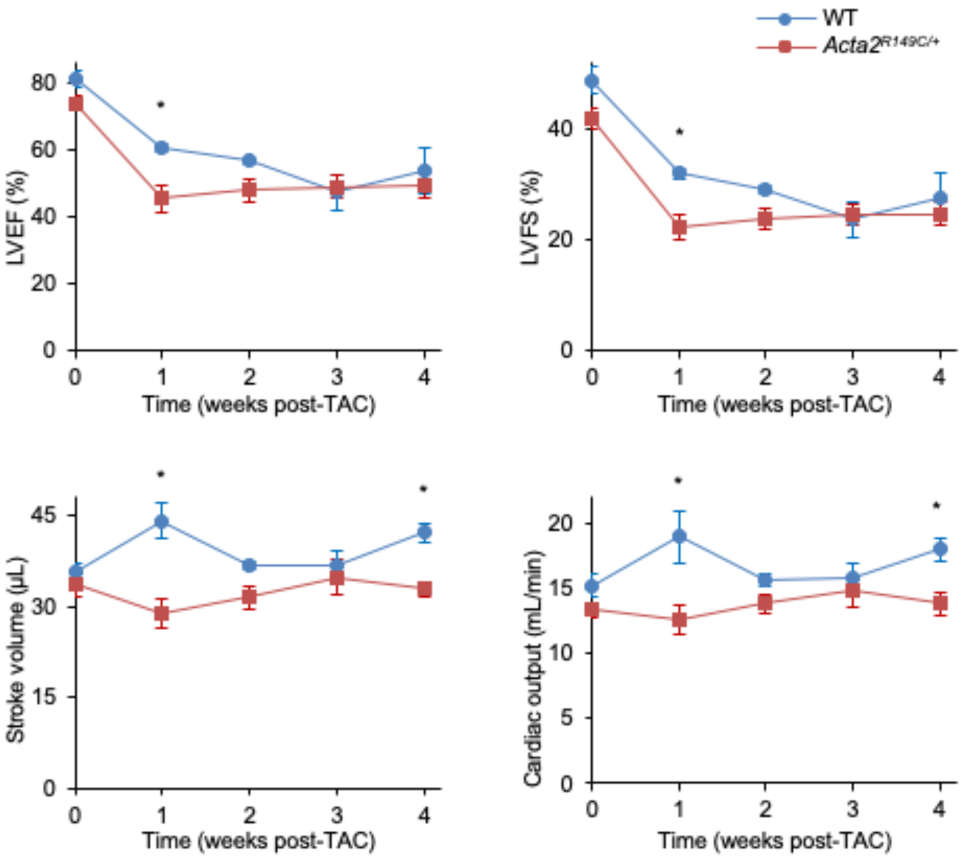


**Supplementary Figure 3: *Acta2$^{R149C/+}$* TAC mice have earlier LV systolic dysfunction than WT TAC mice.** Weekly echocardiograms indicated *Acta2$^{R149C/+}$* mice subjected to trans-aortic constriction (TAC) at 3 months of age had left ventricular systolic dysfunction one week post-TAC compared to WT mice (n=3 or 6 per group, $p<0.05$). Mutant mice followed up to 4 weeks post-TAC had lower cardiac output and stroke volume compared to WT mice ($p<0.05$).

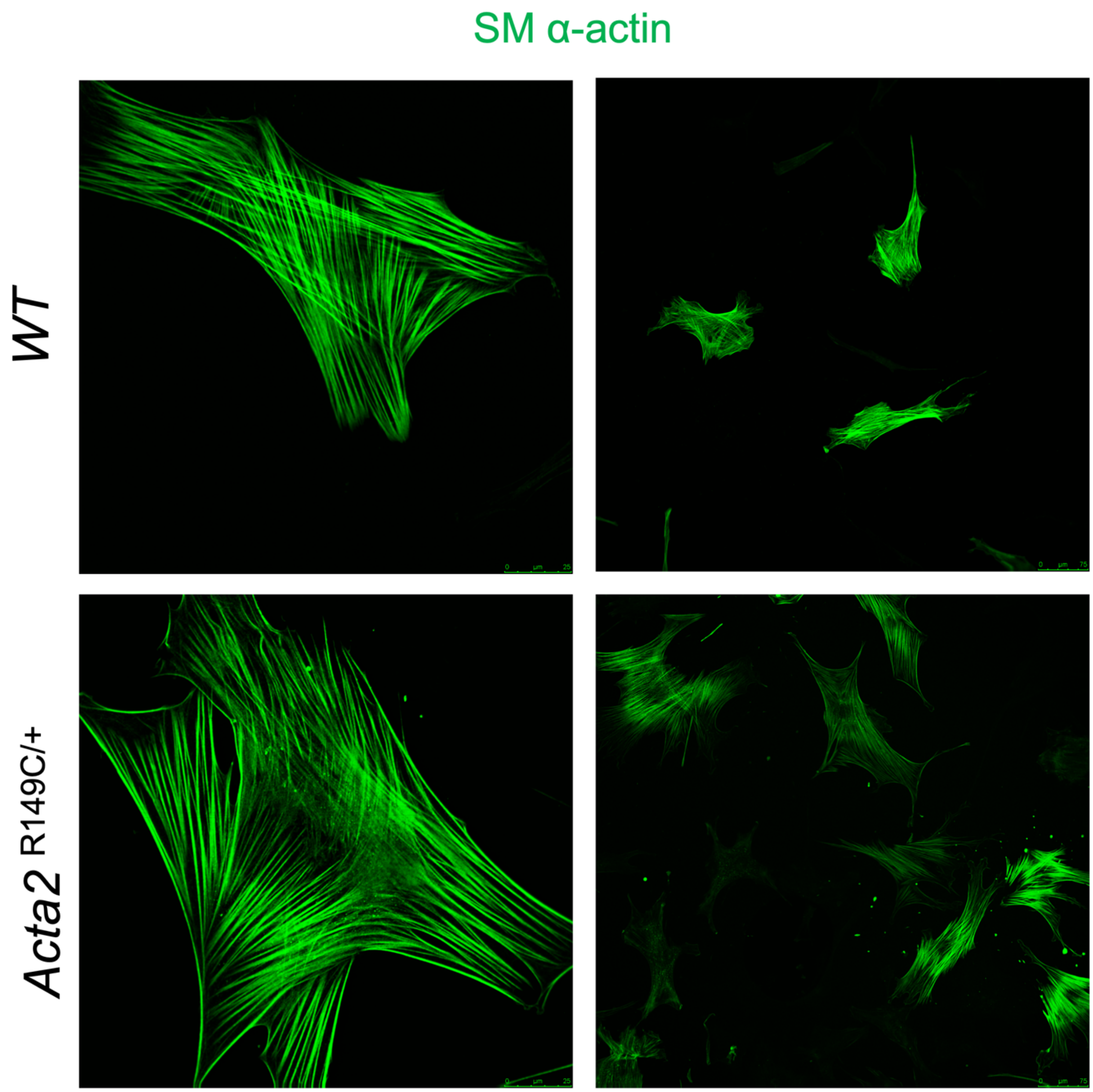


**Supplementary Figure 4: Immunofluorescence of actin filaments.** Immunofluorescence staining using a SM α-actin antibody shows SM α-actin filaments in mutant and WT SMCs. Images were obtained at 10x (scale = 75 μm) and 40x (scale = 25 μm).

**TABLE S1: Quantification of Immunoblot analyses**

| | Calponin | SM α-actin | SM MHC | SM22α |
|---|---|---|---|---|
| **WT aorta** | 0.92 ± 0.32 | 1.52 ± 0.58 | 0.67 ± 0.10 | 0.76 ± 0.20 |
| ***Acta2*$^{R149C/+}$ aorta** | 0.92 ± 0.27 | 1.99 ± 0.52 | 1.42 ± 0.36 | 0.81 ± 0.23 |

[a] Data were obtained from 3 mice per group.
[b] There was a significant increase in SM MHC ($p<0.01$) in mutant mice. There were no significant differences between Calponin ($p=0.97$), SM α-actin ($p=0.17$), and SM22α ($p=0.69$) as determined by two-tailed unpaired t-test. Error are standard deviation (SD).

| | SM α-actin | SM MHC | SM22α |
|---|---|---|---|
| **WT SMCs** | 0.72 ± 0.06 | 0.83 ± 0.10 | 1.21 ± 0.10 |
| ***Acta2*$^{R149C/+}$ SMCs** | 0.63 ± 0.02 | 1.86 ± 0.04 | 0.87 ± 0.09 |

[a] Immunoblot is representative of three repeats.
[b] There was a significant increase in SM MHC ($p=0.02$) in mutant mice. There were no significant differences between SM α-actin ($p=0.25$), and SM22α ($p=0.07$) as determined by two-tailed unpaired t-test. Error are standard deviation (SD).

**TABLE S2. Blood pressures (mmHG) in *Acta2*$^{R149C/+}$ mice versus WT mice with L-NAME and high salt diet (HSD)**

| | SBP Normal diet | SBP L-NAME+HSD | DBP Normal diet | DBP L-NAME+HSD |
|---|---|---|---|---|
| **WT mice**[a] | 120.25 ± 10.97 | 156 ± 14.3 | 88 ± 8.29 | 106 ± 11.63 |
| ***Acta2*$^{R149C/+}$ mice**[b] | 119.25 ± 10.14 | 152 ± 12.57 | 85 ± 9.56 | 104.75 ± 10.34 |

[a]Data were obtained from 8 mice per group. Errors are SD.

**TABLE S3.** ***In vitro*** **motility speeds of movement of actin constructs by phosphorylated smooth muscle myosin.**

| Actin | Phalloidin | Tpm1.4 | Speed (μm/s)[a,b] |
|---|---|---|---|
| **WT** | + | - | 0.796 ± 0.135 (n = 4676) |
| **N299T** | + | - | 0.809 ± 0.130 (n = 2373) |
| **N299T/R149C** | + | - | 0.730 ± 0.152 (n = 3097) |
| | | | |
| **WT** | + | + | 0.940 ± 0.136 (n = 4011) |
| **N299T** | + | + | 0.976 ± 0.128 (n = 1426) |
| **N299T/R149C** | + | + | 0.755 ± 0.167 (n = 1721) |
| | | | |
| **WT** | - | - | 0.691 ± 0.072 (n = 40) |
| **N299T** | - | - | 0.648 ± 0.062 (n = 40) |
| **N299T/R149C** | - | - | 0.467 ± 0.057 (n = 40) |
| | | | |
| **WT** | - | + | 0.882 ± 0.096 (n = 40) |
| **N299T** | - | + | 0.890 ± 0.109 (n = 40) |
| **N299T/R149C** | - | + | 0.775 ± 0.055 (n = 40) |

[a]Speeds of phalloidin–stabilized filaments were determined using a semi-automated tracking program and fitted to a Gaussian distribution as previously described [7]. All pairs (within a set of three) show statistically significant differences (one-way ANOVA followed by a Tukey's Honest Significant Difference post-hoc test) primarily because of the large data set.

[b] Data were obtained using 2 protein preparations and 2-4 individual experiments. For filaments that were not stabilized with phalloidin, WT filaments were formed from 25% rhodamine-labeled WT actin and 75% unlabeled WT. N299T and N299T/R149C filaments were formed from 25% rhodamine-labeled N299T actin and 75% of unlabeled N299T or N299T/R149C actin. Filament speed was tracked manually with ImageJ. All pairs were statistically significant except for WT compared with N299T in the presence of Tpm1.4 (p=0.925) (one-way ANOVA followed by a

Tukey's Honest Significant Difference post-hoc test). Data were obtained using 2 protein preparations and 2 individual experiments.

**Major Resources Table**

**Animals (in vivo studies)**

| Species | Vendor or Source | Background Strain | Sex | Persistent ID / URL |
|---|---|---|---|---|
| Mouse | Jackson Laboratory | C57BL/6NJ | Male/Female | https://www.jax.org/strain/005304 |

**Genetically Modified Animals**

| | Species | Vendor or Source | Background Strain | Other Information | Persistent ID / URL |
|---|---|---|---|---|---|
| **Parent - Male** | Mouse | In house | C57BL/6NJ | *Acta2*$^{R149C/+}$ | |
| **Parent - Female** | Mouse | In house | C57BL/6NJ | *Acta2*$^{R149C/+}$ | |

**Antibodies**

| Target antigen | Vendor or Source | Catalog # | Working concentration | Persistent ID / URL |
|---|---|---|---|---|
| pan-actin antibody | Thermo Scientific | MS-1295-P1 | 0.2μg/mL | https://www.fishersci.ca/shop/products/actin-pan-ab-5-mouse-monoclonal-antibody/ms1295p1 |
| SM MHC | Biomedical Technologies, INC | BT-562 | 0.05μg/mL | https://www.labome.com/product/Biomedical-Technologies/BT-562.html |
| Gapdh | Cell Signaling Technologies | 2118/14C10 | 0.05μg/mL | https://www.cellsignal.com/products/primary-antibodies/gapdh-14c10-rabbit-mab/2118 |
| α-smooth muscle actin (α-SMA) | Millipore Sigma | A5228 | 0.5μg/mL | https://www.sigmaaldrich.com/catalog/product/sigma/a5228?lang=en&region=US |
| SM22α/TAGLN/transgelin | Abcam | ab14106 | 1μg/mL | https://www.abcam.com/taglntransgelin-antibody-ab14106.html |

| | | | | |
|---|---|---|---|---|
| Calponin | abcam | ab46794 | 0.5μg/mL | https://www.abcam.com/calponin-1-antibody-ep798y-ab46794.html |
| pRelA | Cell Signaling | 3033s | 0.05μg/mL | https://www.cellsignal.com/products/primary-antibodies/phospho-nf-kb-p65-ser536-93h1-rabbit-mab/3033 |
| RelA | Cell Signaling | 3034s | 0.05μg/mL | https://www.cellsignal.com/products/primary-antibodies/nf-kb-p65-antibody/3034 |
| pERK1/2 | Cell Signaling | 9101s | 0.05μg/mL | https://www.cellsignal.com/products/primary-antibodies/phospho-p44-42-mapk-erk1-2-thr202-tyr204-antibody/9101 |
| ERK1/2 | Cell Signaling | 9102s | 0.05μg/mL | https://www.cellsignal.com/products/primary-antibodies/p44-42-mapk-erk1-2-antibody/9102 |
| pSmad2 | Invitrogen | 400800 | 0.25μg/mL | https://www.thermofisher.com/antibody/product/Phospho-SMAD2-Ser465-Ser467-Antibody-Polyclonal/40-0800 |
| Smad2 | Invitrogen | 5339s | 0.5μg/mL | https://www.thermofisher.com/antibody/product/SMAD2-Antibody-clone-42-Monoclonal/436500 |
| TCP1 α | Abcam | ab109126 | 0.5μg/mL | https://www.abcam.com/tcp1-alphaccta-antibody-epr4082-ab109126.html |

**DNA/cDNA Clones**

| Clone Name | Sequence | Source / Repository | Persistent ID / URL |
|---|---|---|---|
| ACTA2 WT | NP_001095152.1 | | |
| ACTA2 N299T | NP_001095152.1 | | |
| ACTA2 R149C | NP_001095152.1 | | |
| ACTA2 N299T-R149C | NP_001095152.1 | | |
| ACTA1 WT | BC012597 | DNASU HsCD00301547 | |
| ACTA1 E259V | BC012597 | DNASU HsCD00301547 | |

**Cultured Cells**

| Name | Vendor or Source | Sex (F, M, or unknown) | Persistent ID / URL |
|---|---|---|---|
| Mouse WT SMC | Milewicz Lab, explanted from C57BL/6NJ mice | F, M | |

| | | | |
|---|---|---|---|
| | obtained from The Jackson Laboratory | | |
| Mouse *Acta2*$^{R149C/+}$ SMC | Milewicz Lab, explanted from *Acta2*$^{R149C/+}$ mice in house | F, M | |
